# Supplementary material for: Global analysis of temporal clusters of storm surges
Source: Camb Prism Coast Futur. 2025 Aug 1;3:e17. doi: 10.1017/cft.2025.10008 (PMC12645316; doi:10.1017/cft.2025.10008)
Supplement: Martín et al. supplementary material [file S2754720525100085sup001.docx]

**Global Analysis of Temporal Clusters of Storm Surges**

Ariadna Martín ^1,2^*, Dr. Robert Jane ^1,2^, Dr. Alejandra R Enriquez ^3^ & Dr. Thomas Wahl ^1,2^.

^1^ Department of Civil, Environmental and Construction Engineering, University of Central Florida, Orlando, USA.

^2^ National Central for Integrated Coastal Research, University of Central Florida, Orlando, USA.

^3^ School of Geosciences, College of Arts & Sciences, University of South Florida, St Petersburg, FL 33701, USA

*Corresponding author: ariadna.martinoliva@ucf.edu


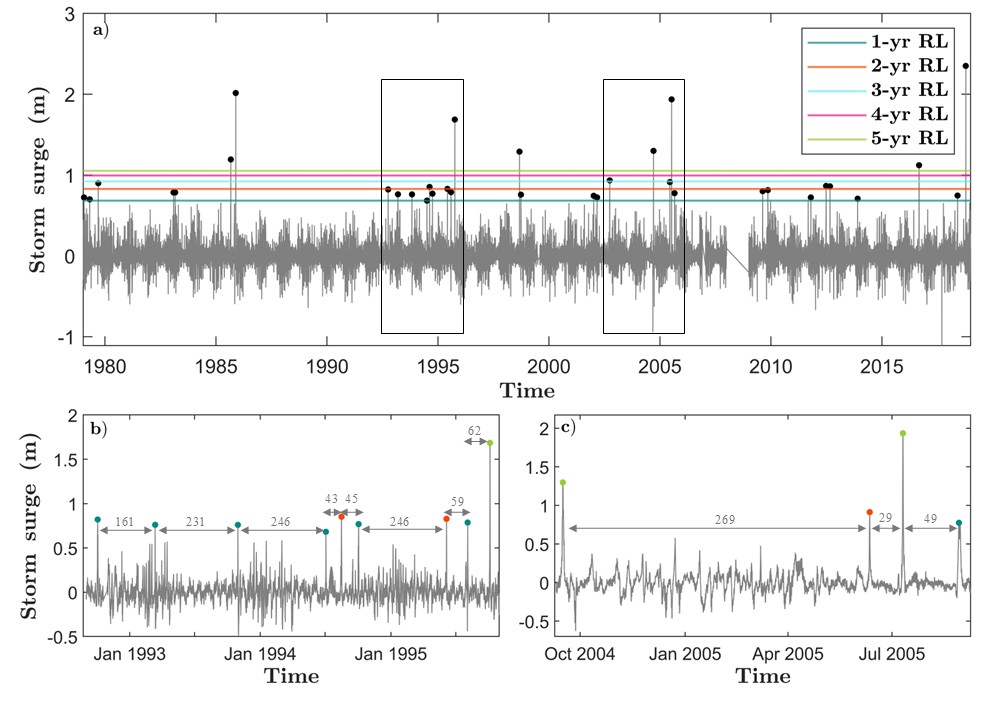


Figure S1. Example of a time series for the Apalachicola tide gauge. Panel a) shows the time series between 1979-2018 (time used for model validation), with the de-clustered events, colors indicate the thresholds. Panels b) and c) show two instances with inter-arrival times. Colors indicate the threshold of the event.


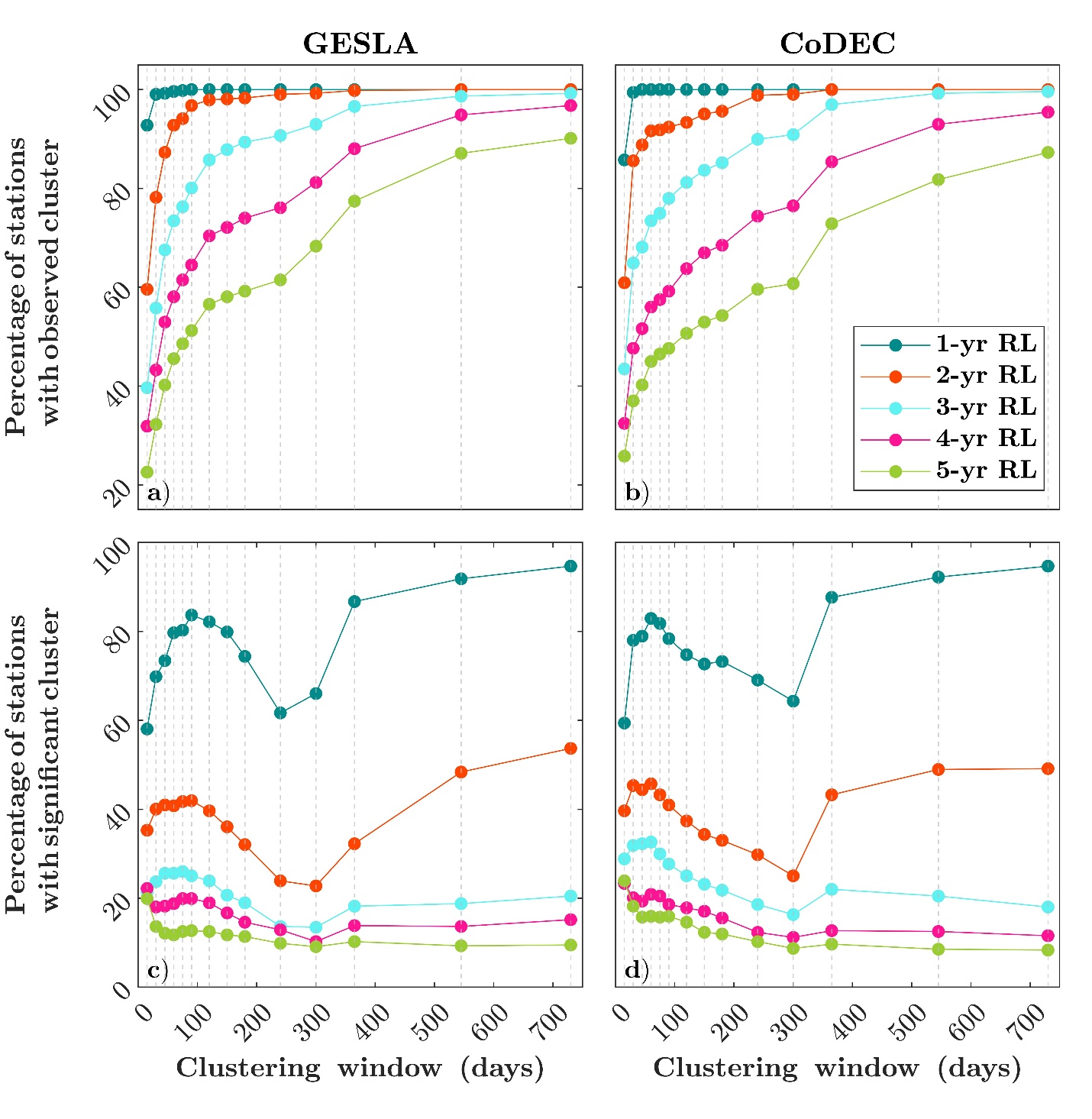


*Figure S2. Identified clusters of skew surges at 527 coastal sites using varying thresholds and clustering windows. a, b. Percentage of locations where clustering occurred based on GESLA (a) and CoDEC (b) data. c, d. Percentage of locations where clustering behavior differs significantly from the assumption of a Poisson distribution for GESLA (c) and CoDEC (d) data.*


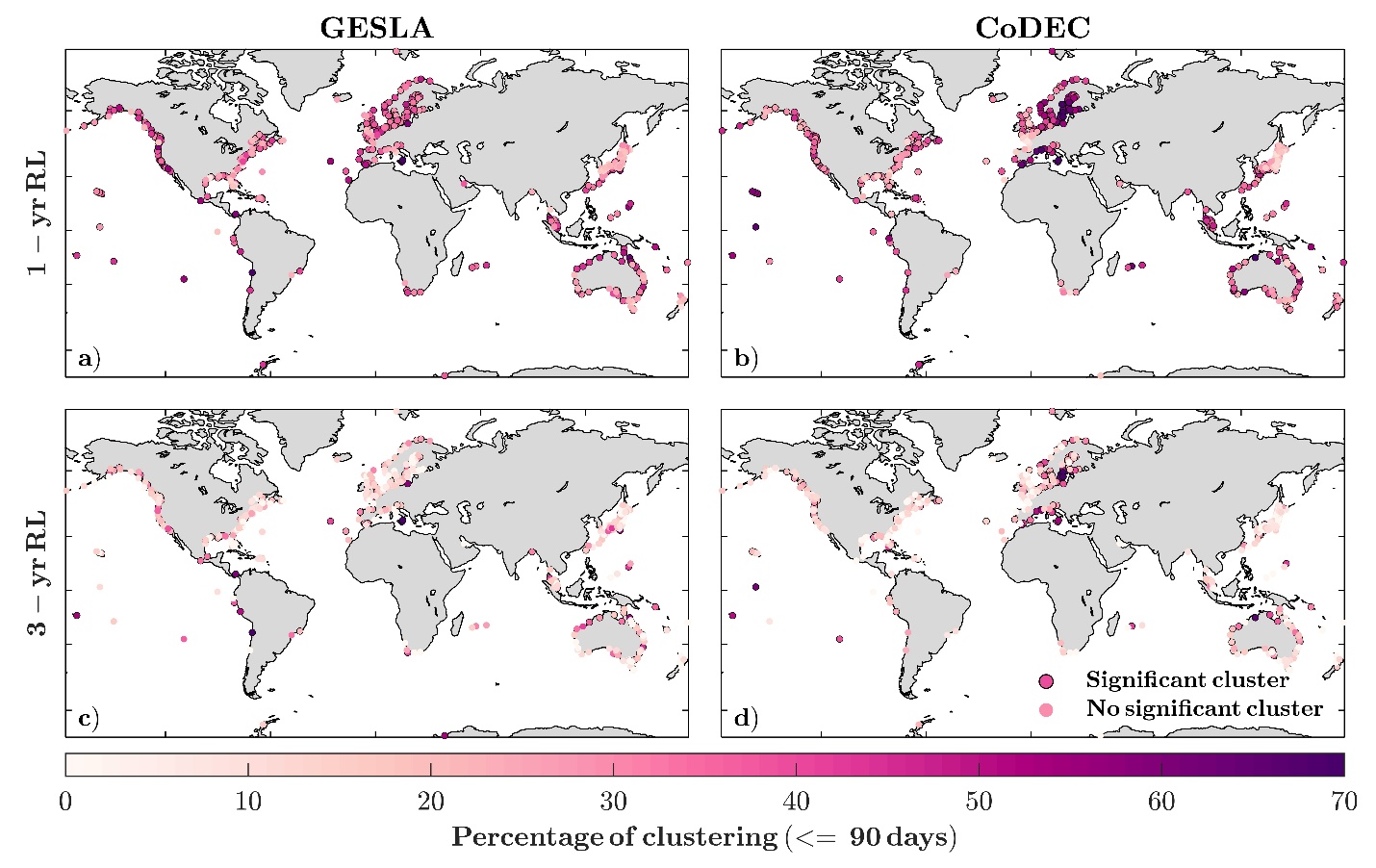


Figure S3. Fig. 3. Percentage of clustering (i.e., number of clusters divided by the number of events) when using a 90-day clustering window and a 1-yr (a, b) and 3-yr (c, d) threshold to identify skew surge events. Results are shown for GESLA (a, c) and CoDEC (b, d) data.


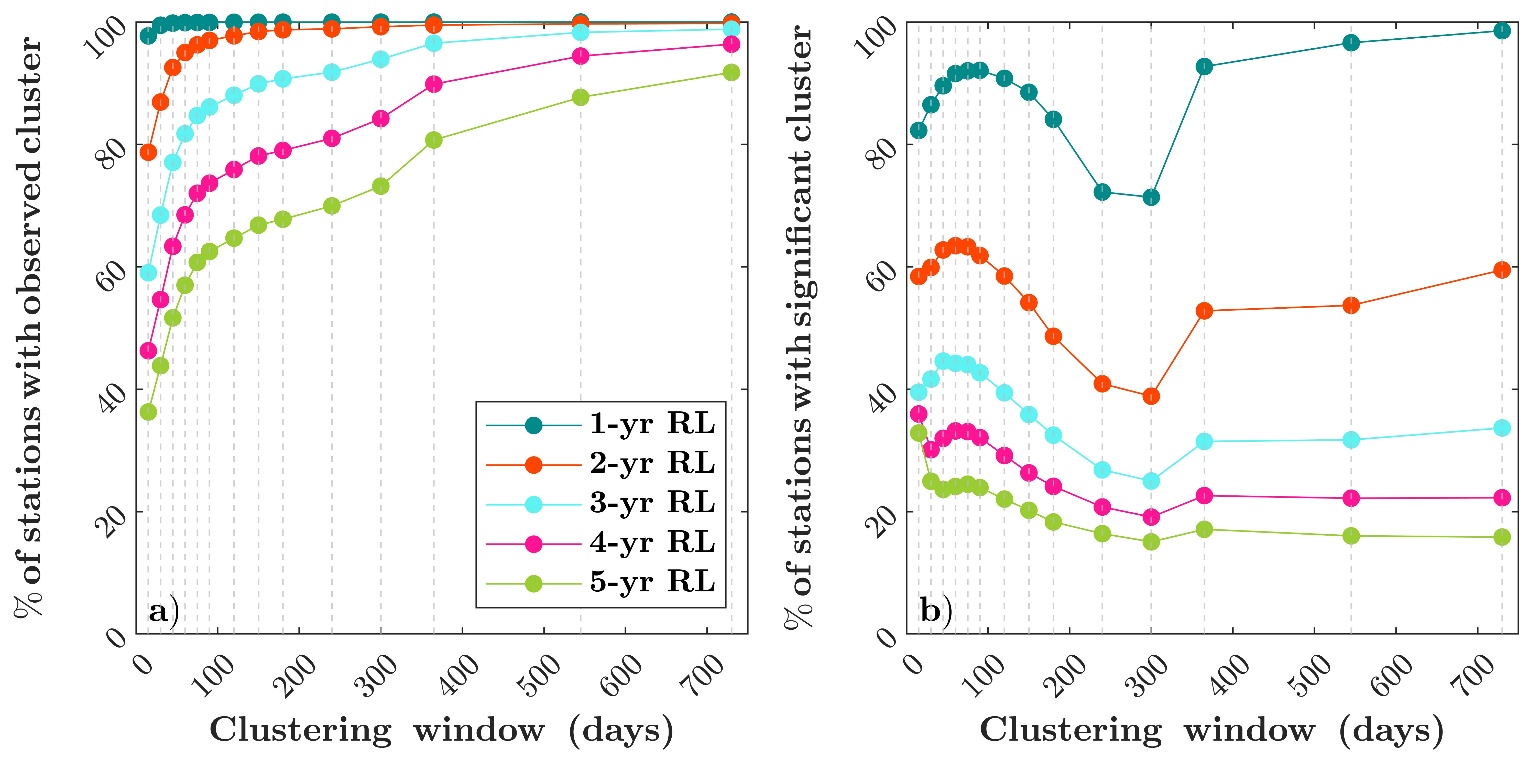


Figure S4. Percentage of observed cluster (a) and statistically significant cluster (b), of all 18,179 CoDEC coastal grid points. Colors indicate different thresholds, and different clustering window definitions (x-axe).


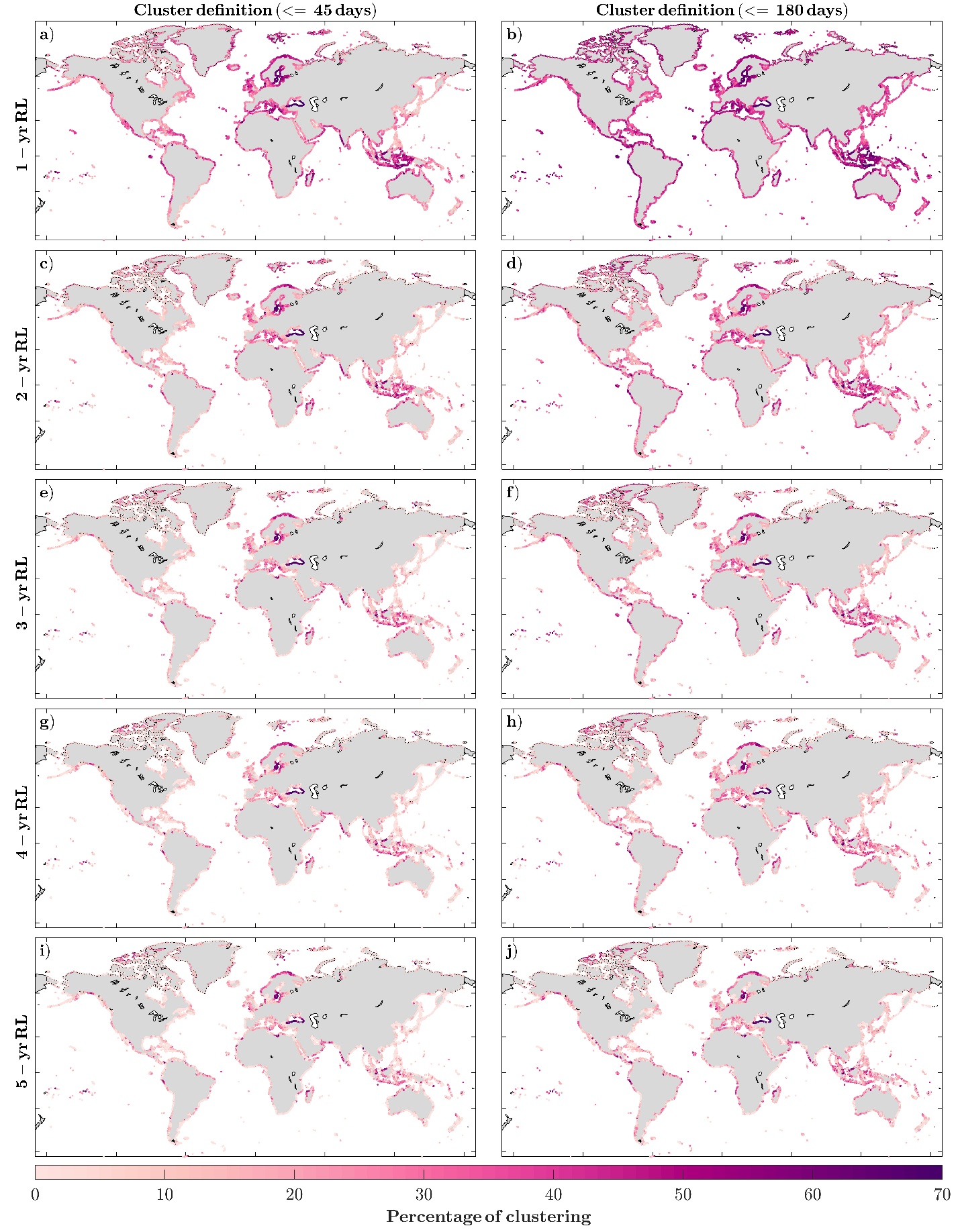


Figure S5. Percentage of clustering, defined as number of clusters divided by the number of events, for clusters of less than 45 days (left-side panels) and 180 days (right-side panels). For events above the 1, 2, 3, 4, and 5-year return levels.


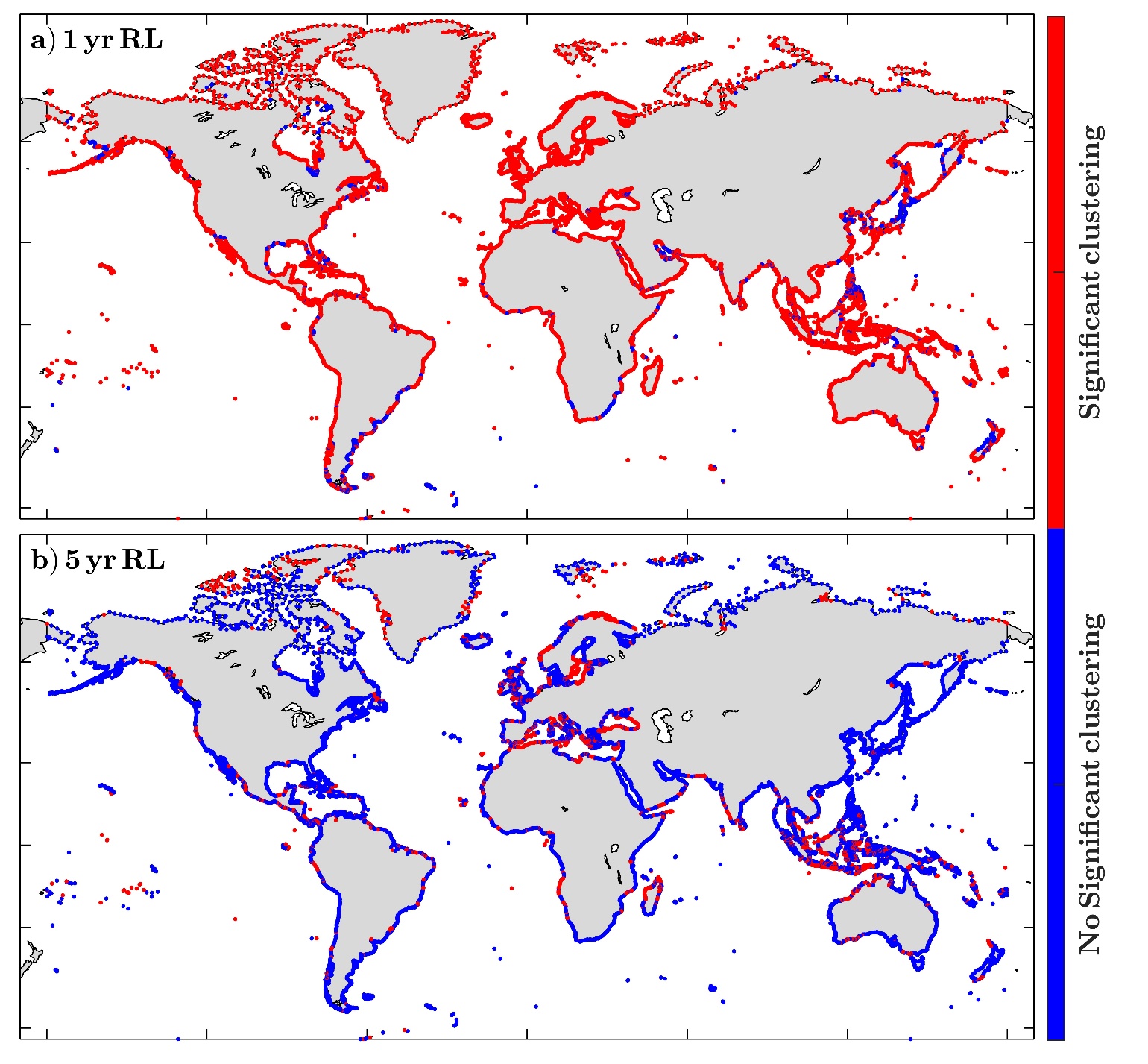


Figure S6. Stations with significant cluster (red) and no significant cluster (blue) for events above a 1-yr return level (a) and 5-year return level (b) of clusters defined as less than 90-days.
